# The complete mitochondrial genome of a species of *Cirrhipathes* de Blainville, 1830 from Kauaʻi, Hawaiʻi (Hexacorallia: Antipatharia)

**DOI:** 10.1080/23802359.2024.2310130

**Published:** 2024-02-01

**Authors:** Leah E. K. Shizuru, Anthony D. Montgomery, Daniel Wagner, Evan B. Freel, Robert J. Toonen

**Affiliations:** a Hawai‘i Institute of Marine Biology, University of Hawai‘i at Mānoa, Kāne‘ohe, HI, USA; b US Fish and Wildlife Service, Pacific Fish and Wildlife Office, Honolulu, HI, USA; c Ocean Exploration Trust, Honolulu, HI, USA

**Keywords:** Black coral, Hawaii, mesophotic coral ecosystem, *Stichopathes*, *Antipathes*

## Abstract

This study reports the first mitogenome from the antipatharian (black coral) genus *Cirrhipathes* (GenBank accession number ON653414). The 20,452 bp mitochondrial genome of *Cirrhipathes* cf*. anguina* LS-2022 consists of 13 protein-coding genes, two rRNA genes, and two tRNA genes (*trnM* and *trnW*). The mitogenome is typical of other antipatharian families, including an A + T biased (64.1%) base composition and cytochrome c oxidase subunit I (*COX1*) intron with embedded homing endonuclease gene (*HEG*). A phylogenetic tree based on complete mitogenome sequences of currently available antipatharians indicates *Cirrhipathes* cf*. anguina* LS-2022 is sister and closely related to *Stichopathes* sp. SCBUCN-8849. However, it seems unlikely that intergeneric taxa share 99.97% similarity across their complete mitogenomes, raising questions about the current taxonomy of this group. This study highlights the need for additional vouchered antipatharian species to be sequenced so phylogenetic relationships can be compared with accepted taxonomy.

## Introduction

Antipatharians, commonly known as black corals, are globally distributed, slow-growing hexacorals (Cnidaria, Anthozoa) that occupy a broad bathymetric range (Wagner et al. 2012; Barrett et al. 2020). This morphologically variable order consists of seven families, 49 genera, and 301 species (Molodtsova and Opresko 2023). Though black corals are found as shallow as 2 m to over 8600 m, more than 75% of species occur below 50 m (Barrett et al. 2020). This predominance presents a logistic challenge to studying this order because most species are located at depths below the limit of conventional Self-Contained Underwater Breathing Apparatus (SCUBA) diving (Wagner 2015). Limited ability to observe living specimens and access to samples leaves these organisms’ basic biology and ecology largely unknown. Although they are less common on shallow-water reefs, antipatharians are essential ecosystem engineers who create habitat for many species of vertebrates and invertebrates in deeper waters (Wagner et al. 2012). In Hawai‘i, black corals are ecologically, culturally, and commercially important, and there has been an active commercial fishery for these corals since 1958 (Grigg 2001) for use in the manufacture of precious coral jewelry. There are three species in the fishery, but current estimates of species composition at harvest depths in the ʻAuʻau Channel (where most harvest occurs) are roughly 93% *Antipathes griggi* and 7% *Antipathes grandis* (Wagner et al. 2017). Black corals are the official gemstone of Hawai‘i and support a $30 million statewide precious coral industry (Grigg 2004), which makes them the focus of considerable management interest for continued sustainable harvest. This mitogenome is the first for this genus and any *Cirrhipathes* cf. *anguina* LS-2022, thus establishing an essential foundation for future studies.

## Materials

**Figure 1.**
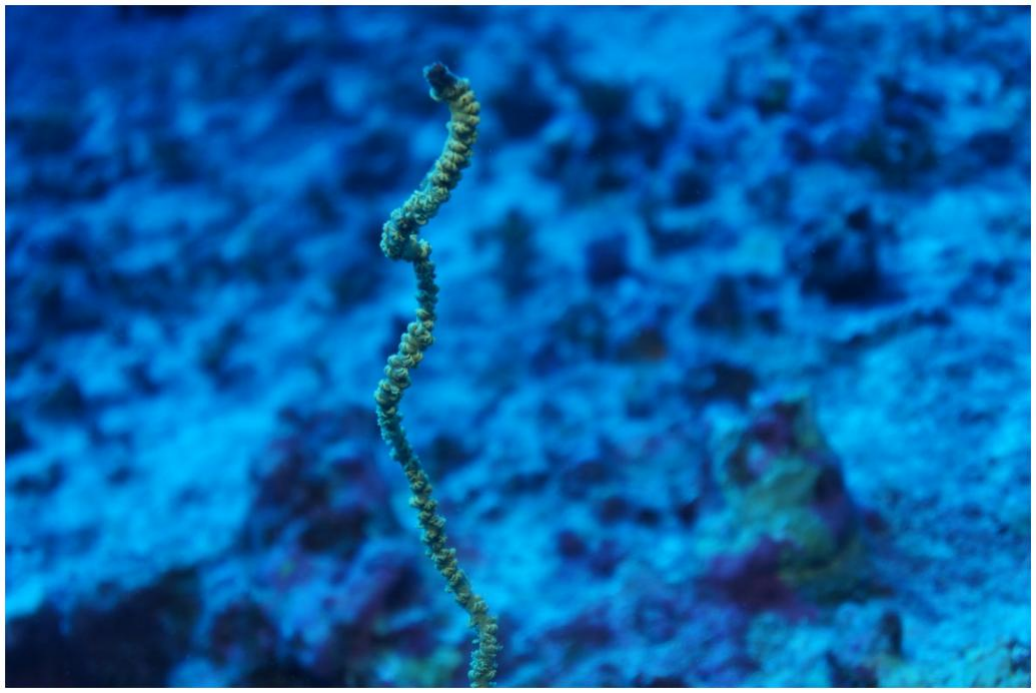
Reference image of *Cirrhipathes* cf. *anguina* LS-2022 taken by Daniel Wagner. Colonies of this wire coral are unbranched and can grow to 2 m or more.

The antipatharian sample used in this study (Figure 1) was collected by hand during a rebreather dive at Amber’s Arches (21.887, −159.602) at 22.86 m off of the island of Kaua‘i and immediately preserved in 95% ethanol. This specimen was deposited at the Bernice Pauahi Bishop Museum (Holly Bollick, holly@bishopmuseum.org, catalogue number: D2772, accession number: 2022.074).

## Methods

**Figure 2.**
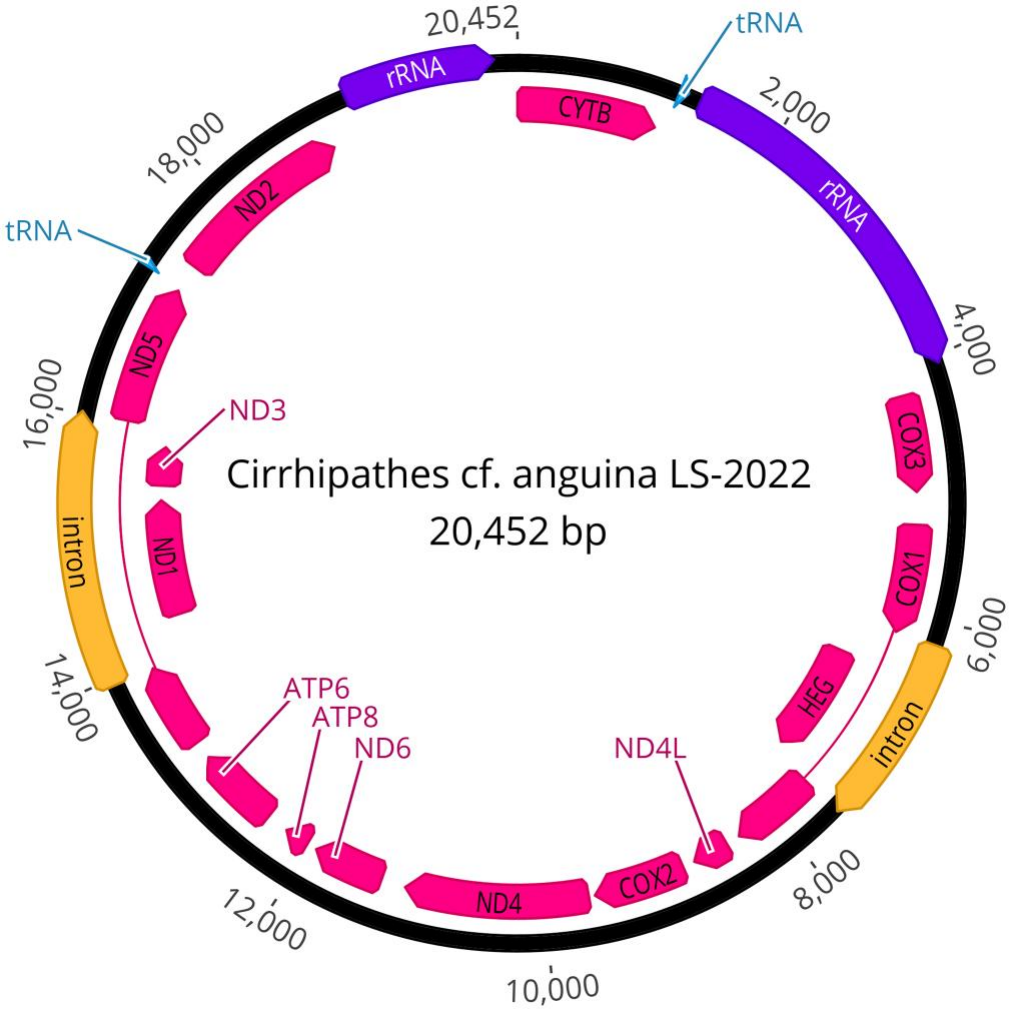
Map of the complete mitochondrial genome of *Cirrhipathes* cf. *anguina* LS-2022, drawn by Geneious Prime version 2022.2.2 (https://www.geneious.com).

**Figure 3.**
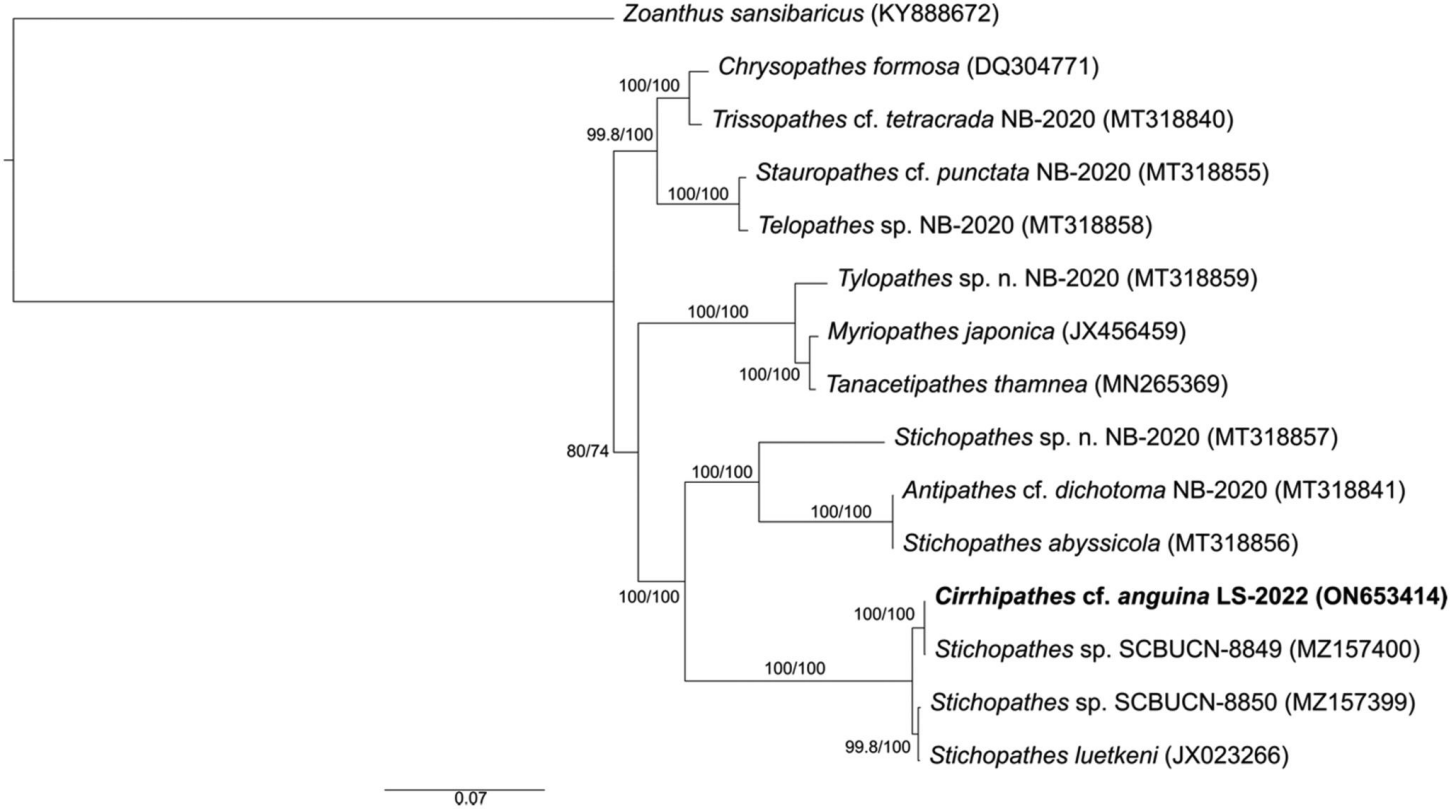
Phylogenetic reconstruction of 13 most closely related antipatharian taxa for which complete mitogenomes are currently available (>87% identical to *Cirrhipathes* cf. *anguina* LS-2022). Branch lengths are relative to genetic divergence, and values on each node represent SH-aLRT/ultrafast bootstrap values. Species used include the following: *Stichopathes* sp. SCBUCN-8849 (Asorey et al. 2021), *Stichopathes* sp. SCBUCN-8850 (Asorey et al. 2021), *Trissopathes* cf. *tetracrada* NB-2020 (Barrett et al. 2020), *Stichopathes* sp. n. NB-2020 (Barrett et al. 2020), *Tylopathes* sp. n. NB-2020 (Barrett et al. 2020), *Antipathes* cf. *dichotoma* NB-2020 (Barrett et al. 2020), *Stichopathes abyssicola* (Barrett et al. 2020), *Telopathes* sp. NB-2020 (Barrett et al. 2020), *Stauropathes* cf. *punctata* NB-2020 (Barrett et al. 2020), *Chrysopathes formosa* (Brugler and France 2007), *Tanacetipathes thamnea* (Figueroa et al. 2019), *Stichopathes luetkeni* (Kayal et al. 2013), and *Myriopathes japonica*.

We sequenced the complete mitochondrial genome of *Cirrhipathes* cf. *anguina* LS-2022 (ON653414) using a restriction-site associated DNA sequencing method (ezRAD, Toonen et al. 2013) on the Illumina platform, an approach documented to recover complete mitogenomes for a variety of coral species (Forsman et al. 2017). Sample identification was based on the original species description (Dana 1846) and identification following Wagner et al. (2010) and Wagner (2015) using gross colony morphology, colony branching pattern, polyp structure, skeletal spine morphology, and scanning electron microscopy of skeletal features. Genomic DNA was extracted using the E-Z 96 Tissue DNA Kit (Omega Bio-Tek, Norcross, GA) with elution in high-performance liquid chromatography (HPLC) grade water. Extracted gDNA was quantified using the Biotium AccuClear Ultra High Sensitivity dsDNA kit. ezRAD libraries were created following the protocol of Knapp et al. (2016). We used the restriction enzyme DpnII (New England Biolabs, Ipswich, MA), and gDNA was size-selected using PCRClean DX (Aline Biosciences, Woburn, MA) beads. As per manufacturer recommendations, DNA fragments of 300–600 bp were prepared for sequencing using the Illumina TruSeq^®^ Nano DNA Library Preparation kit. After passing quality control checks, libraries were sequenced on an Illumina^®^ MiSeq (V3 2 × 300 bp PE). Trim Galore! (Krueger 2015) was used to apply quality filters and trim Illumina adapters from sequence reads. Low-quality base calls (Phred score of <20) were trimmed from the ends before the removal of the first 13 bp of the standard Illumina paired-end adapters ('AGATCGGAAGAGC'). The SPAdes (Bankevich et al. 2012) genome assembler generated a 20,518 bp contig. The contig was circularized, and overlapping ends were trimmed in Geneious Prime 2022.1.1 (https://www.geneioius.com). Protein coding regions were identified via the live annotate feature in Geneious Prime 2022.1.1 (Figure 2) based on published antipatharian mitogenomes from *Stichopathes* sp. SCBUCN-8849 (Asorey et al. 2021), *Stichopathes* sp. SCBUCN-8850 (Asorey et al. 2021), and an updated *Stichopathes luetkeni* JX023266, misidentified initially as *Cirrhipathes lutkeni* (Kayal et al. 2013). Gene annotations were performed using MITOS2 (Bernt et al. 2013). This new mitogenome was then compared to complete antipatharian mitochondrial genomes that were >87% identical to *C.* cf. *anguina* LS-2022 (ON653414) to determine the phylogenetic relationships with closely related taxa except *Zoanthus sansibaricus* (Chi and Johansen 2017) that was chosen as a known outgroup to these species (79.53% similar) (Figure 3). Annotated regions were first extracted from each mitogenome, aligned separately using default settings in the MAFT v7 plugin (Katoh and Standley 2013) for Geneious Prime, and then each annotated region was concatenated into a contiguous sequence following Barrett et al. (2020). The relationship among antipatharians was inferred via maximum likelihood, using IQTREE v. 2.0.3 (Nguyen et al. 2015) under the best-fit substitution model, determined by ModelFinder (Kalyaanamoorthy et al. 2017) for 1000 ultrafast bootstraps (Hoang et al. 2018), as well as the Shimodaira–Hasegawa-like approximate likelihood ratio test (Guindon et al. 2010).

## Results

The mitogenome of *Cirrhipathes* cf*. anguina* LS-2022 is 20,452 bp with a base composition of A (29.0%), T (35.9%), C (15.2%), and G (19.95%). Like other Hexacorallia, there are 13 protein-coding genes, two rRNA genes (*rnl* and *rns*), and two tRNA genes (*trnM* and *trnW*) plus a cytochrome c oxidase subunit I (*COX1*) intron with embedded homing endonuclease gene (*HEG*), as found in other antipatharian families (Barrett et al. 2020, Figure 2). Read depth ranged from 3 to 46, averaging 22 across the mitogenome (Supp. Mat.). The resulting phylogenetic tree places *Cirrhipathes* cf. *anguina* LS-2022 as sister to the closest BLAST hit *Stichopathes sp.* SCBUCN-8849 (Barrett et al. 2020) in our analysis (Figure 3).

## Discussion

*Cirrhipathes* cf. *anguina* occurs throughout the Indo-West Pacific, including the Main and Northwestern Hawaiian Islands at depths of 9–150 m (Wagner 2015). However, initial descriptions are vague, and the type specimen of *C. angina* is lost; therefore, comparisons between *C. anguina* and *C*. cf. *anguina* cannot be made until a neotype is created (Wagner 2015). This species is the first from the genus *Cirrhipathes* to be sequenced to date, but the mitogenome is highly similar (99.97%) to that previously reported for *Stichopathes* sp. (MZ157400, Barrett et al. 2020). Interspecific *Stichopathes* mitogenomes sequenced to date differ by 0.9–2.2%, which is roughly the same magnitude of difference by which these species differ from the mitogenomes of species of *Antipathes* (Asorey et al. 2021). The *Stichopathes* sequenced by Asorey et al. (2021) and our *Cirrhipathes* sample were collected nearly 8000 km apart (Rapa Nui and Hawai‘i) and identified morphologically as belonging to different genera. *Cirrhipathes anguina* is a valid name recognized in the World Register of Marine Species, but intergeneric taxa are unlikely to share similar mitogenomes. Interestingly, reconstructions based on the internal transcribed spacer 1 (*ITS1*) gene place *Stichopathes* sp. SCBUCN-8849 within a clade comprises species within the genus *Cirrhipathes* (Asorey et al. 2021).

This new mitogenome adds to previous phylogenetic work and amplifies the call for additional taxa to be sequenced and for taxonomic revision of the Antipathidae (Bo et al. 2012; Asorey et al. 2021). Phylogenetic reconstructions based on complete mitogenomes are critical to understanding this group’s evolutionary history and taxonomy, which have considerable ecological, cultural, and economic value (Wagner et al. 2012). Additional samples from taxonomically validated *Cirrhipathes* spp. are needed to confirm this sample’s taxonomic affinity relative to congeners and to existing *Stichopathes* samples sequenced across the broad geographic range of the group. Regardless of the taxonomic outcome, this study will provide a basis to evaluate the nominal taxonomy of antipatharians, their geographic distribution, and the relationships among this understudied taxonomic group.

## Supplementary Material

Supplemental Material

## Data Availability

The mitochondrial genome sequence data supporting this study’s findings are openly available in GenBank of NCBI at https://www.ncbi.nlm.nih.gov under the accession no. ON653414. The associated BioProject, SRA, and Bio-Sample numbers are PRJNA868396, SRR21011526, and SAMN29758596, respectively.
