# Supplementary material for: The complete mitochondrial genome of a species of Cirrhipathes de Blainville, 1830 from Kauaʻi, Hawaiʻi (Hexacorallia: Antipatharia)
Source: Mitochondrial DNA B Resour. 2024 Feb 1;9(2):223–6. doi: 10.1080/23802359.2024.2310130 (PMC10836483; doi:10.1080/23802359.2024.2310130)
Supplement: Supplemental Material [file TMDN_A_2310130_SM0017.docx]

**Supplementary Materials**


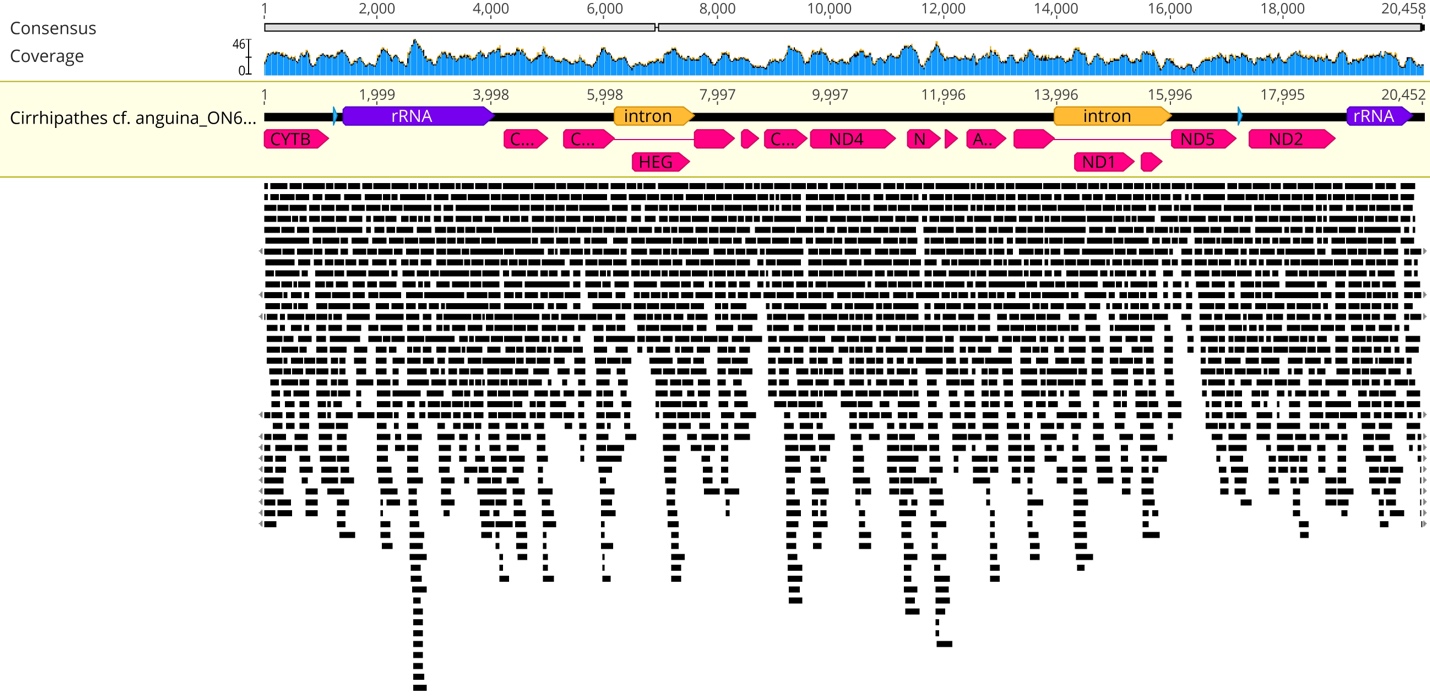


Figure 1. Read coverage depth map of *Cirrhipathes* cf. *anguina* LS-2022 (ON653414) created in Geneious Prime 2022.1.1 (https://www.geneious.com).

**Sample Identification**

Unfortunately, the species description of *Cirrhipathes anguina*, originally collected off Vanua Lebu Island in Fiji, is very brief, with distinguishing characters being yellowish tentacles that are brownish-gray at their base and spines that are laterally compressed and sub-acute (Dana, 1846). These features are evident in the Hawaiian specimen, but the type material for *C. anguina* has been lost, precluding further comparisons. Given that more detailed comparisons can only be made once a neotype is designated, we follow Wagner (2015) and use the name *Cirrhipathes* cf. *anguina* for this Hawaiian wire coral.
